# Facile synthesis of ZnFe_2_O_4_ photocatalysts for decolourization of organic dyes under solar irradiation

**DOI:** 10.3762/bjnano.9.42

**Published:** 2018-02-05

**Authors:** Arjun Behera, Debasmita Kandi, Sanjit Manohar Majhi, Satyabadi Martha, Kulamani Parida

**Affiliations:** 1 Centre for Nano Science and Nano Technology, Institute of Technical Education and Research, Siksha ‘O’ Anusandhan (deemed to be university), Bhubaneswar-751030, India; 2 Department of Electronic Information Materials Engineering, Division of Advanced Materials Science and Engineering, Chonbuk National University, Jeonju, 561-756, Republic of Korea

**Keywords:** Congo red, electrochemical study, phenol, photocatalyst, rhodamine B (Rh B), ZnFe_2_O_4_

## Abstract

ZnFe_2_O_4_ was fabricated by a simple solution-combustion method. The structural, optical and electronic properties are investigated by XRD, TEM, FESEM, UV–vis DRS, PL, FTIR and photocurrent measurements. The photocatalytic activity of the prepared material is studied with regard to the degradation of rhodamine B (Rh B) and Congo red under solar irradiation. The kinetic study showed that the material exhibits zeroth and first order reaction kinetics for the degradation of Rh B and Congo red, respectively. The photocatalytic behaviour of ZnFe_2_O_4_ was systematically studied as a function of the activation temperature. ZnFe_2_O_4_ prepared at 500 °C showed the highest activity in degrading Rh B and Congo red. The highest activity of ZnFe_2_O_4_-500 °C correlates well with the lowest PL intensity, highest photocurrent and lowest particle size.

## Introduction

Photocatalysis is a “green” technology for the treatment of environmental pollutants with solar energy [1]. It is important to develop a suitable environmentally friendly material that can absorb the full range of the solar spectrum for solving energy and environmental challenges. Since last decade, ternary transition metal oxides (TTMOs) have gained tremendous popularity in the field of photocatalysis by the scientific community. To exploit solar light more efficiently, photocatalysts with narrow band gap (e.g., ZnSnO_3_ [2], Bi_2_WO_6_ [3], Ce(MoO_4_)_2_ [4] and ZnFe_2_O_4_ [5]) have been used, for absorbance of solar light in the visible region. Among ternary metal oxides, transition metal ferrites have drawn a great attention in the field of photocatalysis. The reasons are the absorption of a maximum fraction of solar light, and specific optoelectronic and magnetic properties [6–9]. Metal ferrites exhibit a spinel-like structure having the general formula of MFe_2_O_4_ (M = Zn, Cu, Ni, Mn, Co) and are magnetic in nature [10–13]. Among different metal ferrite materials, zinc ferrite (ZnFe_2_O_4_) plays a significant role because of its low band gap (1.88 eV), high thermal conductivity, good chemical stability, higher specific strength, magneto-resistive and magneto optical properties and low fabrication cost [14]. ZnFe_2_O_4_ is an n-type semiconductor having a direct band gap with suitable band edge positions for various photocatalytic processes. It is a solid solution of ferric oxide and zinc oxide, which greatly enhances the charge carrier separation. To date, various methods such as refluxing, hydrothermal [15–17] solid-state combustion, sol–gel, co-precipitation, mechanochemical [18] and microwave-hydrothermal assisted ionic liquids have been reported for the synthesis of metal ferrite [10,13,19]. Recently, Sharma et al. reported the synthesis of ZnFe_2_O_4_ nanoparticles by an urea combustion method that was controlled at 400 °C for 6 h and followed by calcination at 900 °C for 6 h [20]. Yang and co-workers synthesized ZnFe_2_O_4_ nano-octahedrons by one-step hydrothermal reaction at 180 °C for 14 h followed by drying in an oven at 60 °C for 12 h [21]. Dom et al. reported the preparation of ZnFe_2_O_4_ by different physical (SSR and μW) and chemical (PC and SPC) methods with the formation of different structures [22]. Ponhan et al. prepared ZnFe_2_O_4_ nanofibres by electrospinning at room temperature. The resultant ZnFe_2_O_4_/PVP composite nano-fibres were calcined at 500, 600 and 700 °C for 2 h in a furnace at heating and cooling rates of 5 °C·min^−1^ [23]. Most of these preparation processes require various reaction steps or high temperature, which is the major drawback of the prepared materials. The prepared ferrite materials have been widely applied in the field of photocatalysis to degrade most of the organic dyes (i.e., Congo red, Rh B, malachite green methylene blue) to CO_2_, H_2_O and the corresponding mineral acids [19,24]. Movahedi et al. have prepared ZnFe_2_O_4_ through precipitation tested the photocatalytic activity of ZnFe_2_O_4_ in the degradation of 5 ppm Congo red obtaining 64% degradation in 120 min [19]. The photocatalytic activity of ZnFe_2_O_4_ has also been tested in the degradation of Rh B [24]. Zhao et al. have synthesized ZnFe_2_O_4_ through chemical etching followed by calcination and reported a degradation rate of 31% in 3 h [24]. Doong and co-workers have prepared ZnFe_2_O_4_ through a non-aqueous hydrothermal method obtaining 15% degradation of Rh B [25]. The above mentioned reports give an idea about that Rh B and Congo red degradation takes a long time. Hence, our objective is to degrade these harmful organic dyes as fast as possible.

In order to obtain the required phase without impurities, we have synthesized ZnFe_2_O_4_ by a simple solution-combustion method with varying activation temperatures. The effect of the activation temperature has been systematically studied. The property of the material has been examined by various characterisation techniques. Electrochemical studies have been carried out to investigate the photocatalytic decolourization of organic dyes (Congo red and Rh B) under solar light irradiation.

## Experimental

Fe(NO_3_)_3_·9H_2_O (98%, Merck), Zn(NO_3_)_2_·6H_2_O (98%, Lobachemie) and urea (99.5%, Spectrochem) were used directly without any further purification.

### Synthesis of ZnFe_2_O_4_ by solution combustion method

In a typical experiment, 10 mmol of Fe(NO_3_)_3_·9H_2_O, 5 mmol of Zn(NO_3_)_2_·6H_2_O and 30 mmol of urea were collectively added to 30mL distilled water and stirred for 30 min at room temperature. The solution was transferred to a 100 mL alumina crucible and calcined at 500 °C for 10 min in muffle furnace. The calcined sample was cooled to room temperature and ground with mortar and pestle. The obtained powder was calcined at various temperatures such as 400, 500, 600 and 700 °C for 2 h. The samples were subsequently designated as ZFO-400, ZFO-500, ZFO-600 and ZFO-700 [26].

### Photocatalytic decolourization process

The activity in the photo-decolourization of organic dyes such as Rh B and Congo red was evaluated under solar light irradiation for 60 min and 30 min, respectively. The average solar light intensity during the reaction is 100000 lx and the reaction is carried out under broad solar light. For the experiments, 0.02 g of ZFO samples was added to 20 mL of 5 ppm Rh B or 10 ppm Congo red. The obtained suspensions were kept in the dark with stirring for 30 min to develop an adsorption–desorption equilibrium. After that the solutions were exposed to solar light. At regular time intervals, 2 mL of the reaction solution was drawn from the conical flask with the help of a syringe followed by centrifugation to separate the photocatalyst. The solution was then analysed using a UV–vis spectrometer (JASCO 750) at 497 and 554 nm for Rh B and congo red, respectively.

### Sample characterization

The phase composition, crystal structure and crystallinity were examined by a Rigaku Miniflex XRD instrument using Cu Kα radiation (λ = 1.54056 Å). The UV–vis diffused reflectance spectra were analysed by a UV–vis spectrophotometer (JASCO 750) using BaSO_4_ as the reflectance reference. The photoluminescence (PL) emission spectra were investigated by a JASCO-FP-8300 fluorescence spectrometer with an excitation wavelength of 330 nm. The chemical composition and vibrational modes of the ZFO samples were analysed by JASCO FTIR-4600. A ZEISS SUPRA 55 was used for FESEM analysis. Morphology and microstructure of the prepared catalysts were investigated by a TEM-JEOL-2010 200 kV instrument. The electrochemical analysis was carried out using an Ivium potentiostat. Photoelectrochemical (PEC) measurements were performed in a Pyrex electrochemical set up, which includes ZFO samples (deposited on FTO) as working photo anode, Ag/AgCl as reference electrode and Pt as counter electrode. The electrolyte chosen for the study was 0.1 M Na_2_SO_4_ aqueous solution of pH 6.8. A 400 nm cut-off filter was used for the light irradiation during linear sweep voltammetry (LSV) analysis.

## Results and Discussion

### XRD analysis

**Figure 1:**
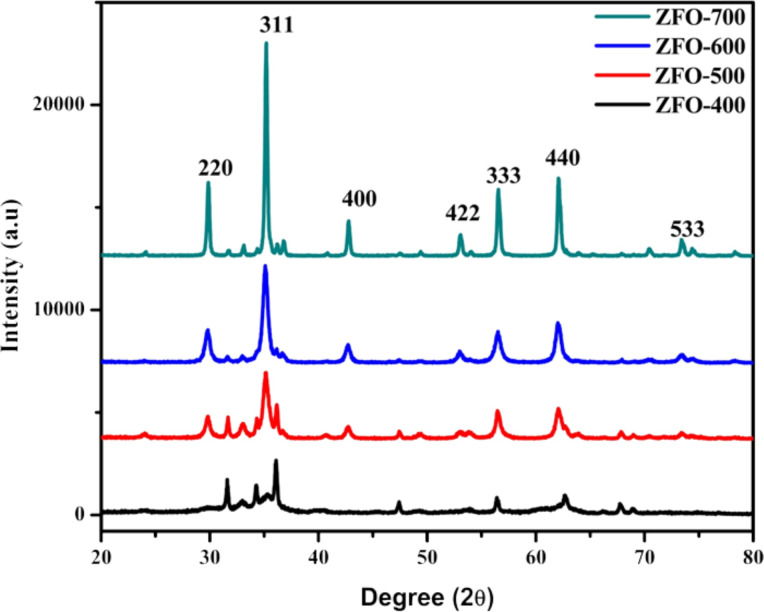
XRD patterns of ZFO samples with different calcination temperatures.

XRD is carried out to identify the crystal structure, phase purity and crystallinity of the materials. Figure 1 presents the XRD patterns of the ZnFe_2_O_4_ samples calcined at different temperatures. The material prepared at 400 °C did not ZnFe_2_O_4_ nanocrystals because the activation temperature is not sufficient. The materials prepared at 500–700 °C are well crystalline and the formation of pure ZnFe_2_O_4_ is confirmed corresponding to JCPDS file no (82-1049). The figure shows strong intensity and narrow peak width, which indicates the formation of crystalline ZFO samples. With increasing reaction temperature, the crystallinity of the prepared ZFO materials increases. The crystallite size has been calculated by using the Scherrer equation, *D* = *K*λ/β cosθ (*K* is a constant having value 0.9, θ is the Bragg diffraction angle, λ is the wavelength of the X-rays used (1.5406 Å) and β is the full-width at half-maximum of the diffraction peak) [27]. The XRD pattern of the ZFO mixed oxide shows peaks at 2θ values of 29.7, 35.1, 42.7, 53.7, 56.4, 62.0, and 73.4^ο^ which correspond to (220), (311), (400), (422), (333), (440) and (533) crystal planes of ZFO crystal structure.

### FESEM

**Figure 2:**
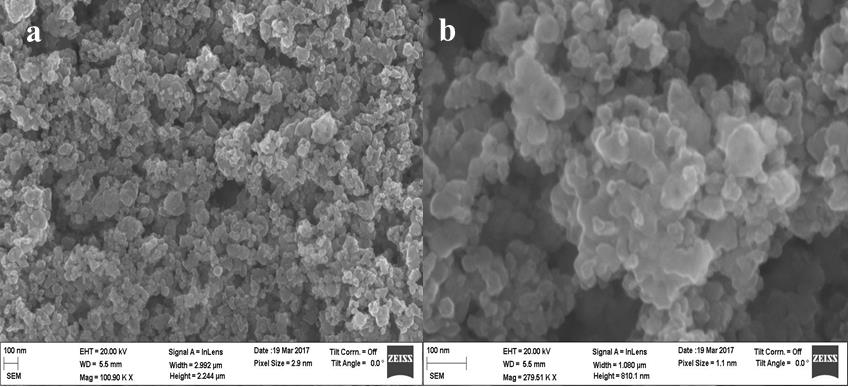
(a) FESEM image and (b) enlarged view of ZFO-500.

Field-emission scanning electron microscopy (FESEM) provides surface morphology and topography of the prepared materials. Figure 2 shows the surface topography of ZFO-500. The sample consists of agglomerated nanoparticles with smooth surface. The agglomeration of ZFO-500 is accompanied with the formation of small crystallites [28].

### TEM

**Figure 3:**
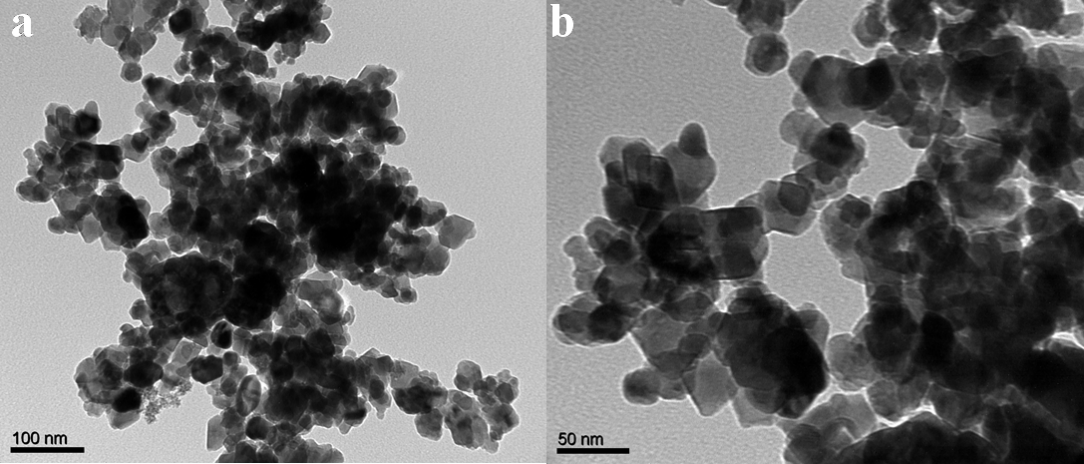
(a) TEM image and (b) magnified TEM image of ZFO-500.

The internal morphology of ZFO-500 was investigated by TEM analysis. The TEM images are shown in Figure 3. ZFO-500 NPs are a mixture of spherical and quasi-spherical particles as shown in Figure 3b. However, most of the ZFO-500 NPs are agglomerated with an average particle size of 50 nm [29].

### UV–vis DRS analysis

**Figure 4:**
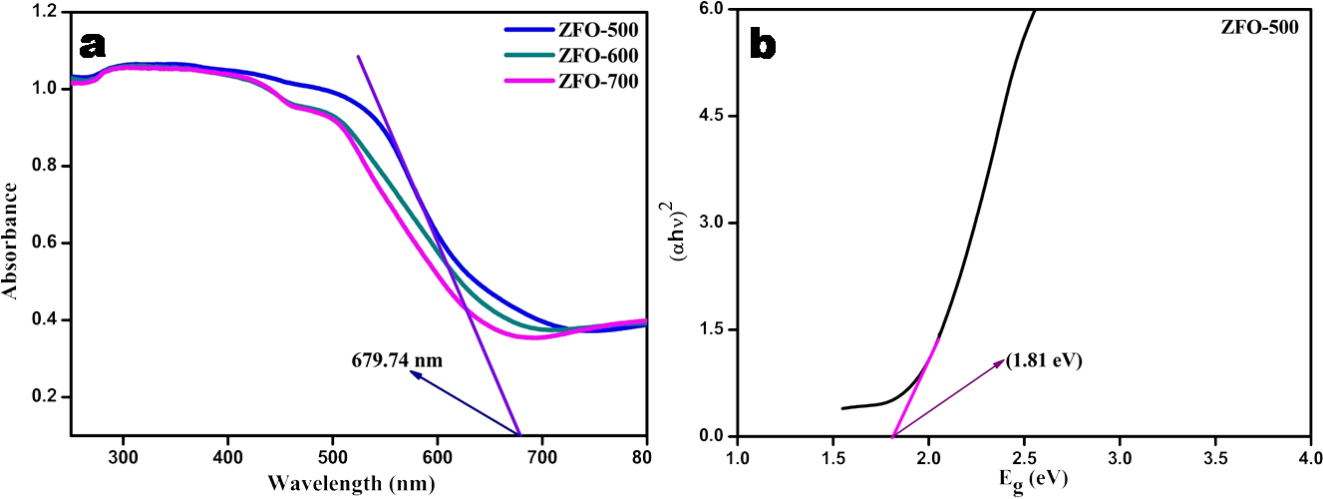
(a) Diffuse reflectance spectra of ZFO samples with different calcinantion temperatures with ZFO-500 showing the highest absorbance; (b) Tauc plot of ZFO-500.

UV–vis diffuse reflectance spectra (DRS) were measured to determine the optical absorption properties of the ZFO samples. The samples show a wide absorption range from 200 to 700 nm as depicted in Figure 4a, and among them ZFO-500 shows the highest absorption. With increasing calcination temperature the absorption of the materials shifted towards higher wavelengths. The band-gap energy of ZFO-500 has been calculated with the help of Tauc plots using Equation 1 [30]:

[1]
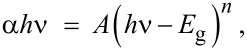


where α, *h*, ν, *E*_g_, and *A* are absorption coefficient, Plancks constant, frequency of light, band-gap energy and a proportionality constant, respectively. In the above equation, n determines the transition in a semiconductor, i.e., *n* = 1/2 for a direct transition and *n* = 2 for an indirect transition. Figure 4b shows that the band-gap energy of ZFO-500 C is 1.81 eV.

### PL analysis

**Figure 5:**
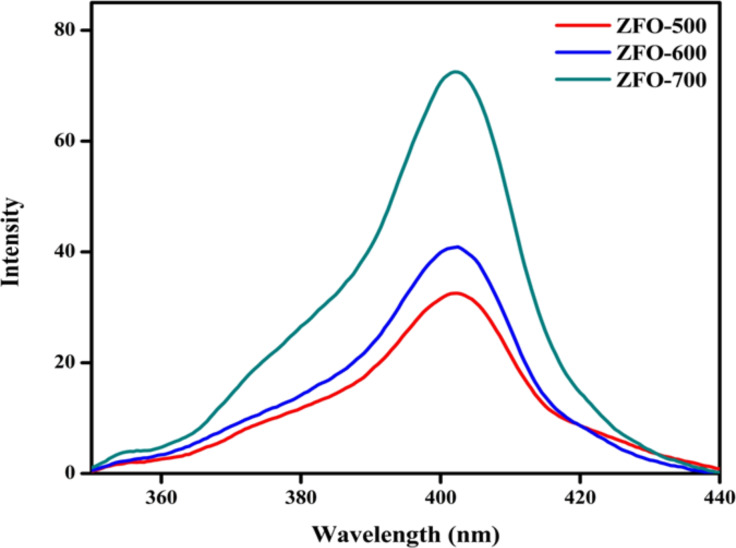
Photoluminescence spectra of ZFO samples excited at 330 nm.

The PL emission can be used to investigate the charge separation process and the fate of photoinduced electron–hole pair. Figure 5 shows the photoluminescence spectra of the ZFO samples calcined at different temperatures. The most intense PL band of ZFO is centred around 400–410 nm when excited at 330 nm. PL spectra with low-emission intensity correlate with a low recombination rate of photo-excited charge carriers and vice versa [31].

ZFO-700 exhibits an intense peak indicating higher electron–hole recombination rate. ZFO-500 exhibits low PL intensity indicating a high electron–hole separation efficiency. It can be concluded that ZFO-500 should possess the highest photocatalytic activity, which is in good agreement with the experimental photocatalytic activity (degradation of Congo red and Rh B) discussed later.

### FTIR analysis

**Figure 6:**
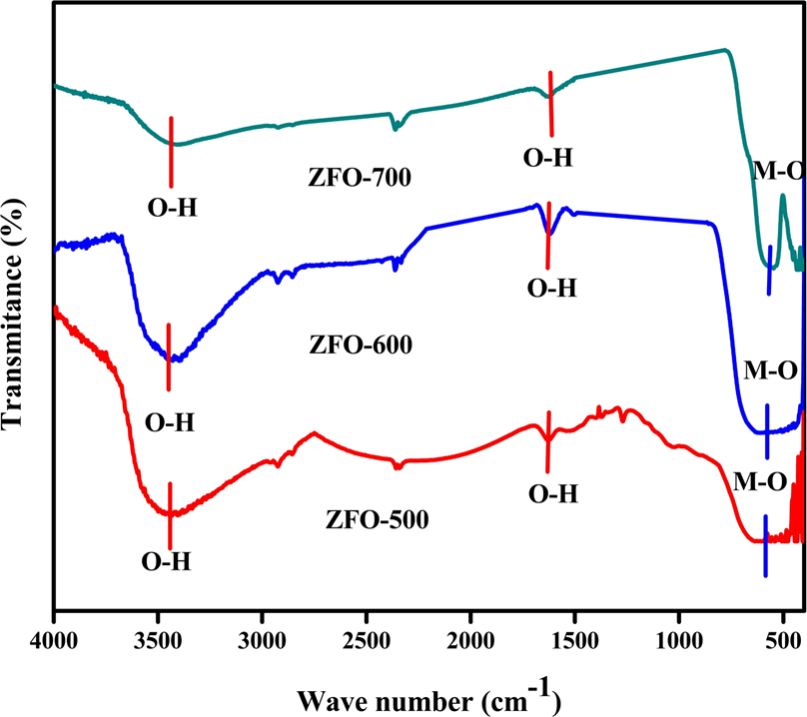
FTIR spectra of ZFO samples.

FTIR spectra of the ZFO samples were measured in the range of 4000–400 cm^−1^ (Figure 6). The bands in the region of 3600–3300 cm^−1^ and in the region of 1650–1550 cm^−1^ (red lines) represent the stretching vibration and deformation vibration of surface-adsorbed hydroxy groups. The peak centred at 2350 cm^−1^ is characteristic for anti-symmetrical stretching mode of dissolved carbon dioxide [32]. The stretching band in the region of 590–540 cm^−1^ is due to stretching vibrations of M–O bonds (Zn-O and Fe-O).

### Electrochemical studies

#### Linear-sweep voltammetry

**Figure 7:**
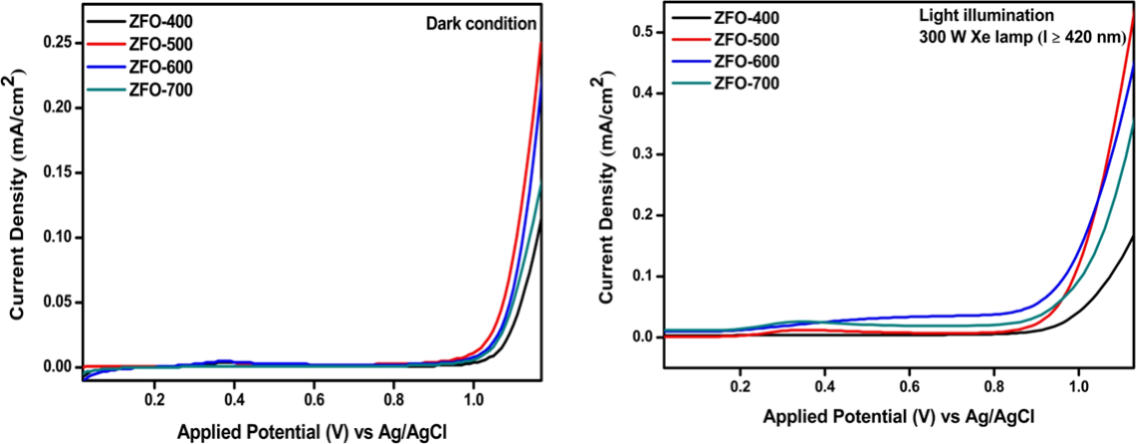
Linear-sweep voltammograms of ZFO-400 to ZFO-700 (a) under dark conditions, (b) under visible-light illumination.

In order to know the photocurrent response of the prepared catalysts, linear-sweep voltammetry was carried out in the range of 0–1.1 V in 0.1 M Na_2_SO_4_ at a scan rate of 10 mV·s^−1^. Figure 7 shows photocurrent measurements. Under dark conditions, ZFO-500 could generate a current of 0.25 mA/cm^2^. Under light illumination of the rear side of ZFO-coated FTO, the current is significantly increased up to 0.54 mA/cm^2^.

#### Mott–Schottky analysis

Mott–Schottky analysis was performed to obtain the band edge of the prepared materials. The Mott–Schottky graphs were plotted according to Equation 2:

[2]
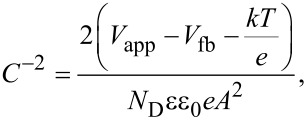


where *C*, *V*_fb_, *k*, *T*, *e*, *N*_D_, ε, ε_0_ and *A* are capacitance of the sample, flat-band potential, Boltzmann constant, absolute temperature, electron charge, donor density, semiconductor dielectric constant, dielectric constant in vacuum and area, respectively. *C*^−2^ was plotted as a function of the applied potential, *V*_app_. The extrapolation of the graph leads to the intersection point at the *Y*-axis, which gives the flat-band potential of the sample [33].

**Figure 8:**
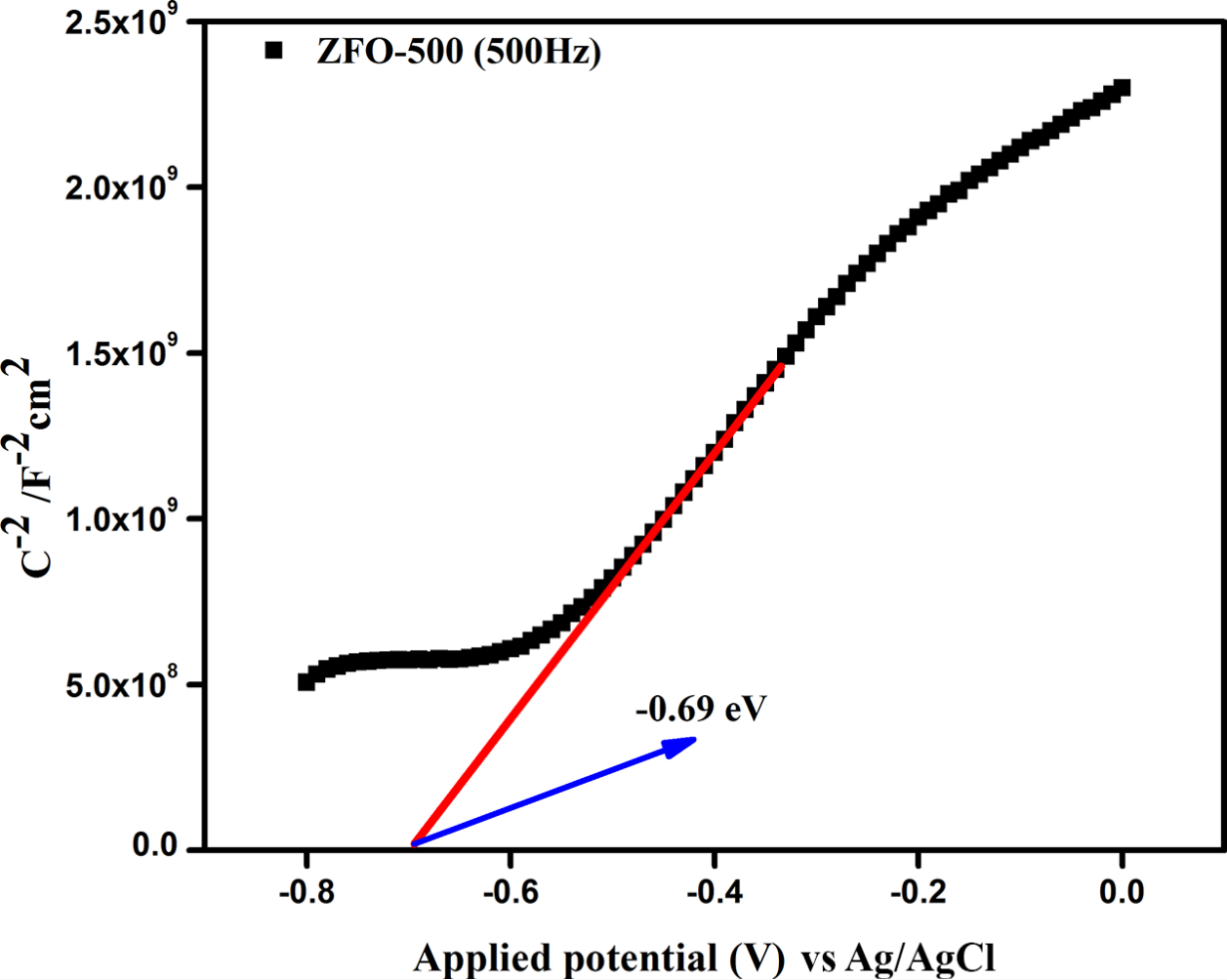
Mott–Schottky plot yielding the flat-band potential of ZFO-500.

Figure 8 shows the Mott–Schottky plot of ZFO-500. The material is an n-type semiconductor and the flat-band potential (*E*_fb_) was calculated to be −0.69 eV (vs Ag/AgCl) or −0.09 eV (vs RHE). From UV–vis DRS measurements, the band gap of was found to be 1.81 V. So, the valence band position of ZFO is calculated as +1.72 eV (vs RHE). By considering the calculated band-edge potential, a mechanism of the photocatalytic reaction has been proposed which is discussed later.

#### Electrochemical impedance study

**Figure 9:**
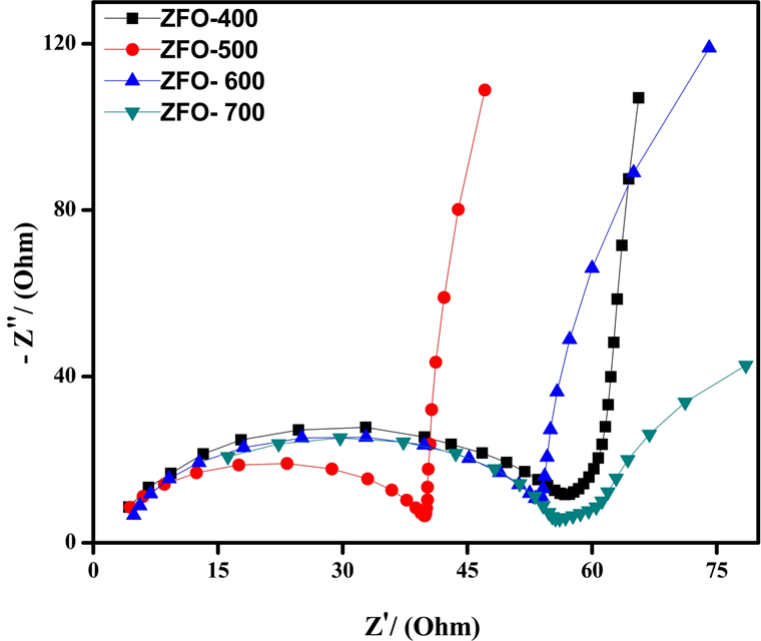
Nyquist plot of ZFO-400 to ZFO-700 photo anode in a frequency range from 50 to 10^5^ Hz.

Impedance measurements are commonly used to determine the charge transfer, resistance, and effective charge separation processes occurring at electrode–electrolyte interface. In general, the radius of the semicircle in the Nyquist plots yields information about the resistance and interfacial charge migration, i.e., a smaller radius reflects a better flow of charge or low interfacial resistance compared to larger radii. ZFO-500 offered the fastest interfacial charge transfer, effective charge separation and high conductivity (Figure 9). This result is well supported by PL measurements and photocatalytic activity.

### Photocatalytic activity

#### Decolourization of Congo red under solar-light irradiation

**Figure 10:**
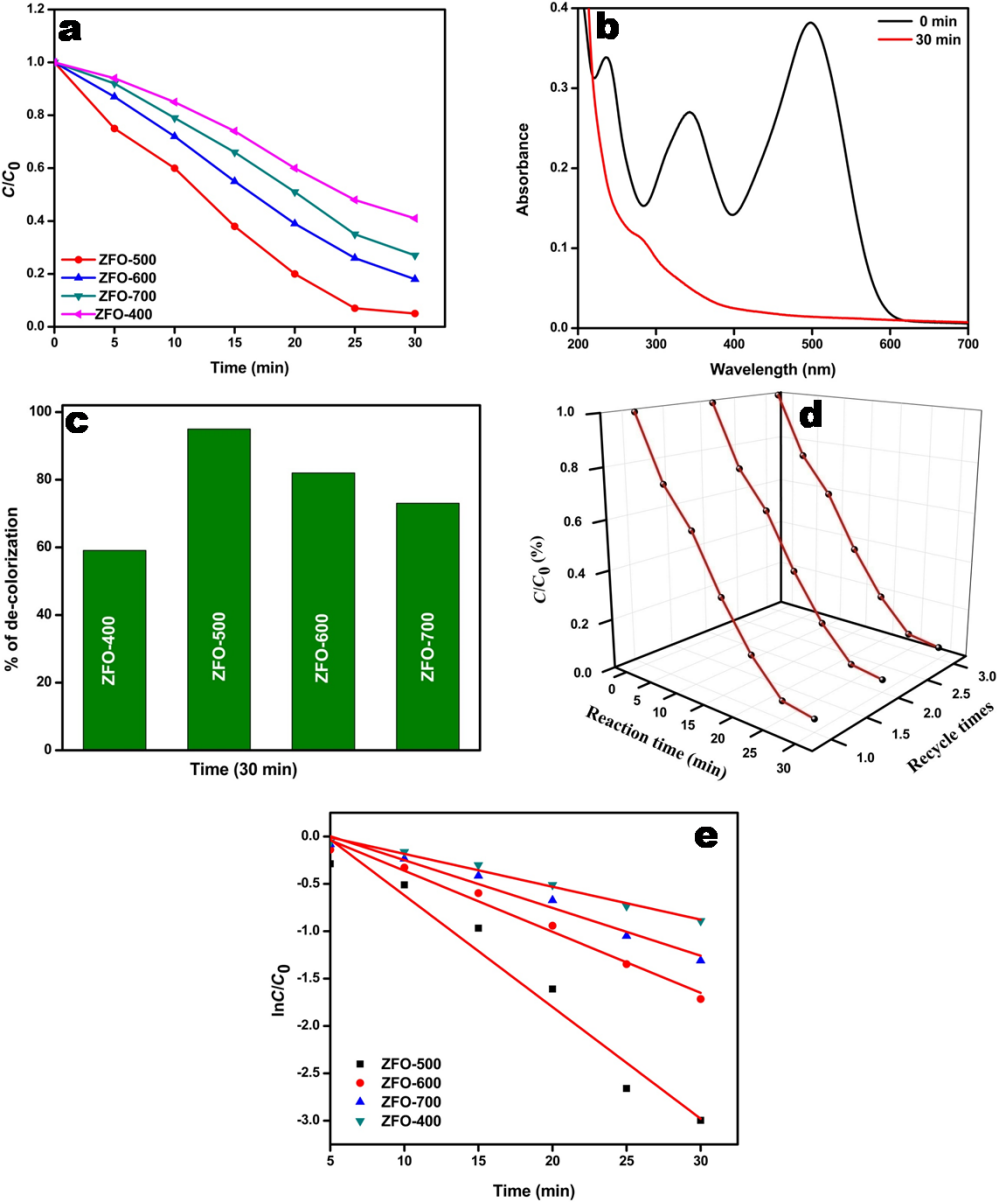
(a) Photocatalytic decolourization of Congo red over different ZFO samples; (b) spectral changes of the Congo red solution over ZFO-500; (c) rate of decolourization of Congo red over various ZFO samples and (d) repeated stability test of the photocatalytic activity of ZFO-500; (e) kinetics linear fit in this process.

The photocatalytic activity in the decolourization of 10 ppm Congo red (CR) under solar irradiation was determined. To know the self-degradation of the dye, a control experiment was carried out by exposing Congo red solution to the light source for 30 min. The results showed that the Congo red solution could not be photo-degraded in the absence of the photocatalyst. In the next step, the adsorption ability of the catalyst was studied under dark conditions for 30 min. It was confirmed that the adsorption effect of the catalyst is negligible regarding the decolourization of a Congo red solution. Then 0.02 g of photocatalyst was used to decolourize 20 mL of 10 ppm of Congo red solution under solar irradiation for 30 min. The decolourization rate of Congo red solution is presented in Figure 10a and the corresponding spectral changes are represented in Figure 10b. A decolourization of 95% was obtained in the first 30 min with ZFO-500 (Figure 10c), more than with the other prepared materials. The order of decolourization efficiency is ZFO-500 > ZFO-600 > ZFO-700 > ZFO-400. For ZFO-400, ZFO-600 and ZFO-700 the decolourization rate is 59%, 82% and 73%, respectively (Figure 10c). ZFO-500 has a higher stability (up to three cycles as shown in Figure 10d) than the other ZFO samples. The results for decolourization activity are in good agreement with the results obtained from the other measurements.

The values of BET surface area of ZFO-400, ZFO-500, ZFO-600 and ZFO-700 are 34 m^2^/g, 17 m^2^/g, 14.5 m^2^/g, 6.9 m^2^/g. This result shows that the surface area has no effect towards photocatalytic degradation process [34].

#### Kinetics of the decolourization of Congo red

The kinetics involved in the decolourization of Congo red are shown in Figure 10e. The data obtained from the experiment was fitted with the model for first-order reactions, i.e., ln *C*_0_/*C* = *kt*) and the fitted parameters are given in Table 1.

**Table 1:** First-order fitting results of Congo red decolourization over ZFO samples.

catalyst	*R*^2^	*k*_obs_ (min^−1^)	*t*_1/2_ (min)	% decolourization after 30 min

ZFO-400	0.83	34·10^−3^	20.38	59
ZFO-500	0.97	117·10^−3^	5.92	95
ZFO-600	0.93	64·10^−3^	10.82	82
ZFO-700	0.90	50·10^−3^	13.86	73

#### Scavenger test for the radicals involved in the process

**Figure 11:**
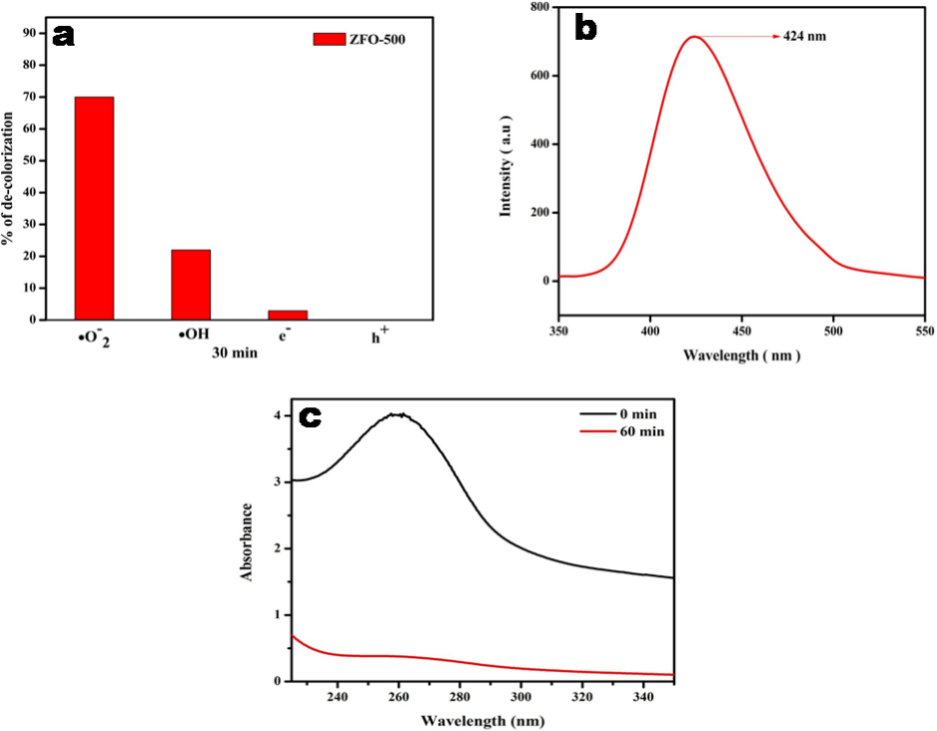
(a) Histogram representing the role of active species in the decolourization of Congo Red; (b) fluorescence spectra of ZFO-500 solution with TA; (c) absorbance spectra of nitroblue tetrazolium solution with ZFO-500 before and after experiment.

In order to analyse the mechanism of degradation, the most active species formed during the decomposition of Congo red were examined by scavenging experiments. The active species responsible for the decolourization of Congo red can be detected by using trapping reagents such as dimethyl sulfoxide, isopropyl alcohol, ethylenediaminetetraacetic acid, *p*-benzoquinone for electrons, hydroxyl radicals (•OH), holes (h^+^), and superoxide (•O_2_**^−^**) radicals, respectively [35]. Figure 11b,c clearly shows that hydroxyl and superoxide radicals play a major role in the decolourization of Congo red solution. When isopropyl alcohol, a •OH scavenger, was added to the Congo red solution, the rate of decolourization was decreased. When *p*-benzoquinone was added, the same behaviour was observed. Thus, it can be concluded that •OH and •O_2_**^−^** are the active species in the decolourization process. The confirmation of the active species is studied in next section.

#### Confirmatory test for •OH radical

**Scheme 1:**
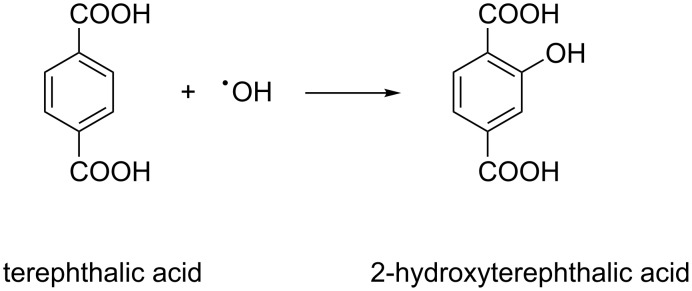
Formation of luminescent 2-hydroxyterephthalic acid from terephthalic acid.

In order to confirm the major role of •OH radicals, terephthalic acid (TA) was utilised as a probe reagent because it does not react with other radicals and molecules in the solution (•O_2_**^−^, •**HO_2_, H_2_O_2_). TA readily reacts with **•**OH and forms 2-hydroxyterephthalic acid (HTA) via the reaction shown in Scheme 1.

In a typical procedure, 5 mM of TA was mixed with the required amount of catalyst and an equimolar ratio of NaOH, as TA is not soluble in acidic or neutral medium. The whole solution was then exposed to solar light for 30 min, and then PL analysis was carried out. HTA can be detected by PL measurements. It shows emission at λ = 426 nm under excitation at λ = 315 nm, while TA does not fluoresce. The emission peak at 424 nm in Figure 11b proves the formation of HTA. The more **•**OH radicals are formed, the higher will be the intensity of the PL peak. The results confirm that **•**OH radicals are the active species in the decolourization of Congo red over ZFO samples.

#### Confirmatory test for •O_2_^−^ radicals

In order to confirm the formation of •O_2_**^−^** radicals, a 5·10^−5^ M solution of nitroblue tetrazolium (NBT) was taken as molecular probe. 0.01 g of ZFO was dispersed in 10 mL of the prepared NBT solution and irradiated under solar light for 1 h. After reaction, the catalyst was separated and the solution analysed in an UV spectrophotometer. Figure 11c shows the results and it suggests the generation of •O_2_**^−^** radical through ZFO-500. By the end of reaction the concentration of NBT was found to be decreased which clearly indicates the formation of •O_2_**^−^** during the decolourization process. This confirms the contribution of •O_2_^−^ in the decolourization of Congo red.

#### Decolourization of rhodamine B under solar light irradiation

**Figure 12:**
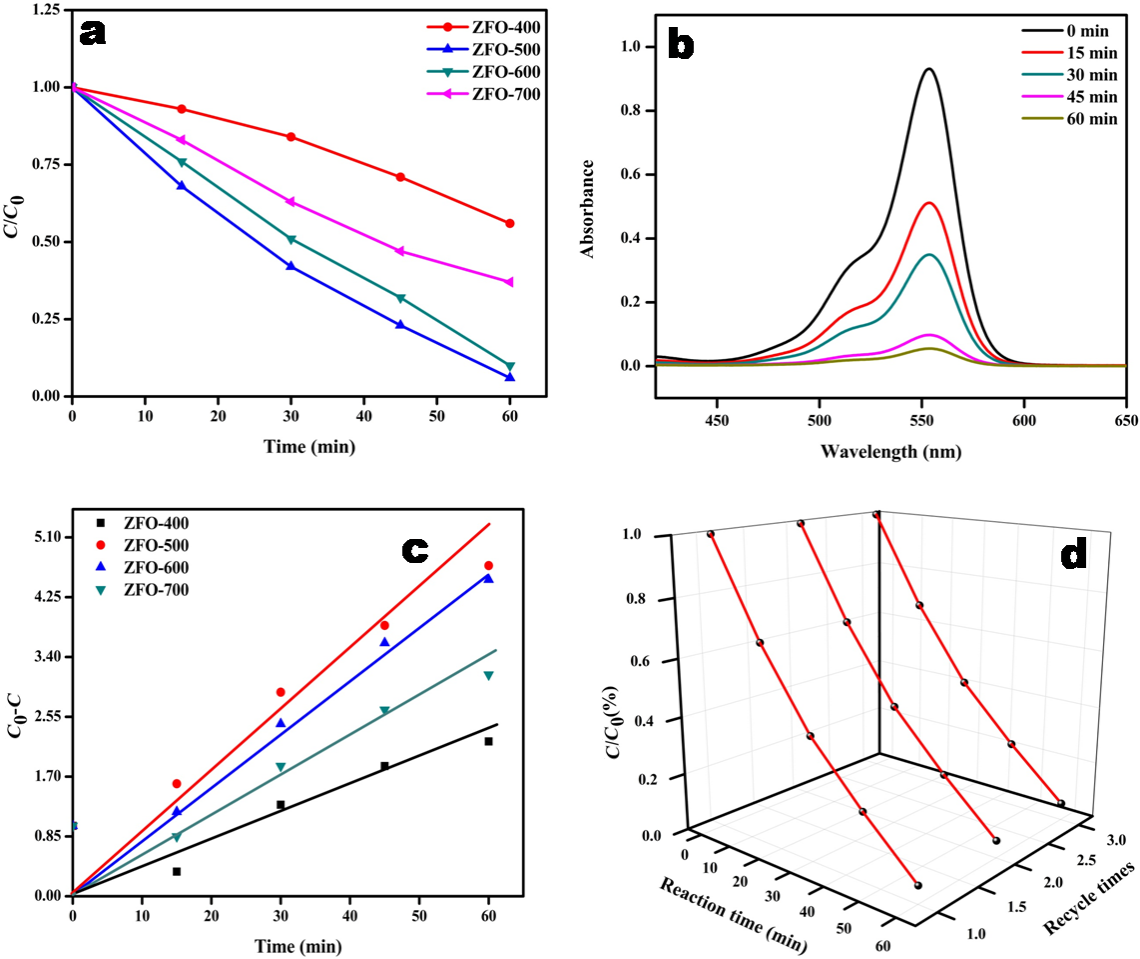
(a) Rate of decolourization of 5 ppm Rh B over ZFO samples; (b) spectral changes of Rh B over ZFO-500 after different time intervals; (c) zero-order kinetics plot of Rh B decolourization over ZFO samples; (d) stability graph of ZFO-500.

The photocatalytic activity of as prepared ZFO samples was also tested in the decolourization of Rh B. ZFO-500 has also proven to be a good photocatalyst in decolourization of 5 ppm Rh B in 1 h. The experimental procedure was similar to that in the decolourization of Congo red. The results show that among the prepared ZFO samples, ZFO-500 could decolourize 93.6% in 1 h. The percentage of decolourization over all ZFO samples follows the order ZFO-500 (94%) > ZFO-600 (90%) > ZFO-700 (62%) > ZFO-400 (43%). The decolourization as a function of the time is presented in Figure 12a. Figure 12b shows the absorption spectrum of the Rh B solution containing ZFO-500 as function of the time.

The obtained resulted data indicates that decolourization process follows zero-order kinetics (Figure 12c) and the derived parameters are listed in Table 2. The zero-order rate constant for ZFO-500 is found to be 0.064 mg·L^−1^min^−1^ which is lowest among all ZFO samples. The stability was also tested by repeating the decolourization experiment using the same ZFO-500 sample. ZFO-500 could retain its activity up to three cycles (Figure 12d).

**Table 2:** Zero-order fitting parameters of Rh B decolourization over ZFO samples.

catalyst	*R*^2^	*k*_obs_ (mg·L^−1^·min^−1^)	*t*_1/2_ (min)	% decolourization

ZFO-400	0.84	0.026	96.15	43
ZFO-500	0.98	0.064	39.06	94
ZFO-600	0.95	0.062	40.32	90
ZFO-700	0.90	0.040	62.5	62

#### Proposed mechanism for the decolourization of Congo red and Rh B

**Figure 13:**
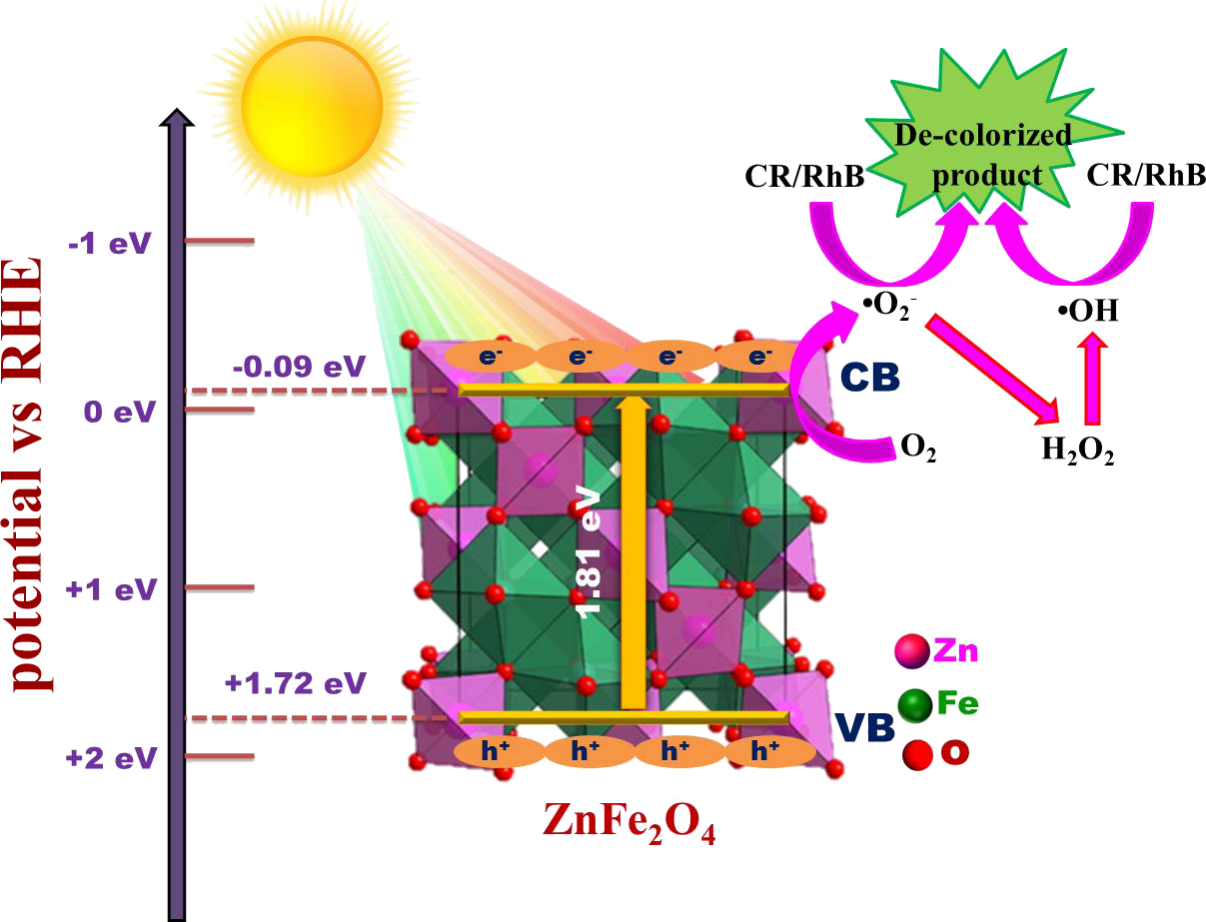
Reaction mechanism of the decolourization of Congo red and Rh B over ZFO-500.

Based upon the above results, the mechanism of the photocatalytic decolourization of Congo red and Rh B is illustrated in Figure 13. Electron and holes generated in ZFO are the agents for the production of the main active species in the photodegradation. CB and VB position of ZFO were determined at −0.09 and +1.72 eV, respectively, from the Mott–Schotky plots. The conduction band of ZFO is more negative than *E*_0_ of •O_2_**^−^** (−0.046 eV vs NHE) [36]. Hence, electrons from the CB of ZFO are able to produce **•**O_2_**^−^** from dissolved oxygen. According to the scavenger tests, **•**OH radicals play an important role [37]. However, from the band gap positions of ZFO it can be derived that the direct formation of **•**OH is not possible from the photogenerated holes since *E*_0_ of •OH/OH^−^ = +1.99 eV vs NHE. Instead, the radicals are generated indirectly from H_2_O_2_. The entire decolourization process can be described by the following equations:


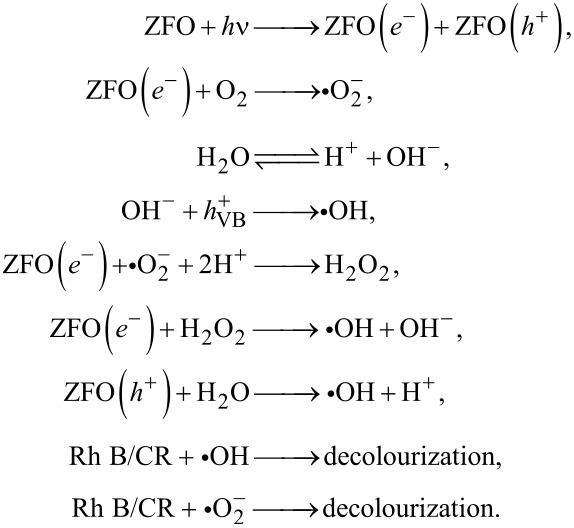


Hence the net reaction is Rh B/CR + **•**OH + **•**O_2_**^−^** → decolourization.

#### Degradation of phenol

**Figure 14:**
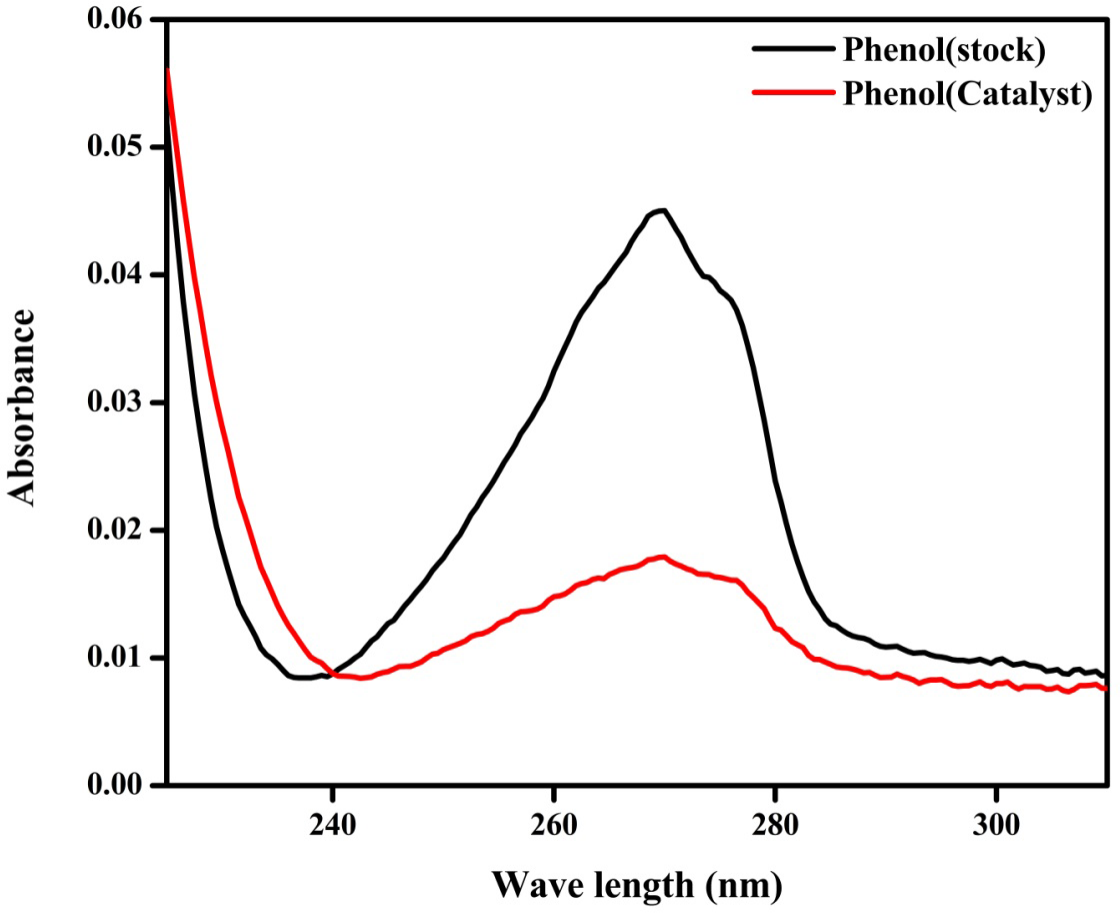
Adsorption spectra of the phenol solution before and after photodegradation over ZFO-500.

In order to show its versatility, the ZFO-500 photocatalyst was also tested in degradation of colourless organic pollutants. We have used phenol as a model pollutant. The photocatalytic degradation rate of 10 ppm phenol was found to be 60% after 60 min over ZFO-500. The degradation procedure of phenol is similar to the degradation of Rh B and Congo red [35]. Photodegradation of phenol was performed by using 20 mL of a 10 ppm phenol solution containing 0.02 g of photocatalyst in a 100 mL conical flask kept under dark conditions for 30 min to ensure adsorption–desorption equilibrium. Then the photo degradation reaction was carried out for 1 h under 250 W visible light and stirring for 60 min. Then the catalyst was removed and the residual phenol was analysed with JASCO 750 UV–vis spectrophotometer. Figure 14 shows the spectral changes after degradation for 60 min. Utilizing the Beer–Lambert law, the degradation was found to be 60% for ZFO-500.

## Conclusion

ZnFe_2_O_4_ photocatalysts were successively fabricated by a facile solution-combustion method. XRD showed the formation of cubic ZnFe_2_O_4_. TEM showed the particles are spherical with an average size of 50 nm. PL study reveals that ZnFe_2_O_4_-500 has lowest PL intensity, which means the electron–hole recombination is minimum leading to the generation of a high photocurrent. From the degradation study it is concluded that ZFO-500 is able to degrade Congo red and Rh B. The active species **•**OH and **•**O_2_**^−^** play a major role in the photocatalytic decolourization process. This work will provide new ways for the further improvement of semiconductor materials for energy and environmental applications.
